# Continuous Tracking and Recognition of Small Objects in Video Streams Based on YOLO and Spatio-Temporal Contextual Memory Networks

**DOI:** 10.3390/s26144639

**Published:** 2026-07-22

**Authors:** Chengyuan Pang, Zongpu Li, Le Ru, Fan Sun, Jiaxu Chen

**Affiliations:** 1 Equipment Management and Unmanned Aerial Vehicle Engineering School, Air Force Engineering University, Xi’an 710051, China; 15082073451@163.com (C.P.); ru-le@163.com (L.R.); 19929246729@163.com (F.S.); 18993762301@163.com (J.C.); 2 National Key Laboratory of Unmanned Aerial Vehicle Technology, Xi’an 710051, China; 3 The Youth Innovation Team of Shaanxi University, Xi’an 710051, China

**Keywords:** YOLOv8, spatio-temporal context, memory network, video stream, small objects, continuous tracking and recognition

## Abstract

**Highlights:**

**What are the main findings?**

- The proposed method, which integrates WPConv, CBAM, STCM-Net, BiFPN, and the Inner-PIoU loss function into a YOLOv8n-based framework, significantly improves continuous tracking and recognition metrics for small objects, achieving a MOTA of 0.99 and a MOTP of 0.98.
- The method maintains a high success rate for the continuous tracking and recognition of various small object categories even under different conditions of occlusion.

**What are the implications of the main findings?**

- The method provides an effective solution for high-precision, persistent small-object tracking in complex video stream scenarios, addressing key challenges like small pixel proportion, feature loss, and occlusion.
- The ablation study demonstrates the contribution of each proposed module, offering a modular and extensible architectural improvement paradigm for YOLO-based models in video analysis tasks.

**Abstract:**

Small objects in video streams occupy a small proportion in the image; the texture and shape information they carry is limited, making it difficult to continuously track and identify. To solve this problem, a method for continuous tracking and recognition of small objects in the video stream based on YOLO and spatio-temporal context memory network is proposed. A backbone network based on the improved YOLOv8 model is introduced, and the multi-scale visual features of small objects are extracted at different levels of the video stream using the wavelet pooling module. A mixed attention module enhances the feature response in the spatially significant pixel regions, generating weighted multi-scale visual features. The neck network processes these weighted features through a spatio-temporal context memory network to extract multi-scale spatio-temporal features. Then, a bidirectional feature pyramid module fuses these multi-scale spatio-temporal features. The head network processes the fused features to output continuous recognition results for small objects. Experiments show that the proposed method successfully extracts the spatiotemporal features of small objects from video stream data sets dominated by small objects. Under different conditions of small object occlusion rates, this method achieves a success rate of continuous tracking and recognition of small objects over 0.93.

## 1. Introduction

Currently, rapid advancements in fields such as intelligent surveillance and drone navigation have generated massive volumes of video stream data [1]. As one of the core tasks in computer vision, object tracking and recognition play a pivotal role within this context: in intelligent surveillance, it facilitates the timely detection of abnormal target behaviors, enhancing the timeliness and effectiveness of security protection [2]; in drone navigation, it empowers drones with autonomous, continuous tracking capabilities for dynamic targets, thereby expanding their application scope [3]. However, small targets in video streams—such as distant pedestrians or minute obstacles—occupy a minimal proportion of the image, contain limited feature information, and are susceptible to external factors like lighting variations and occlusion interference. This frequently leads to tracking loss or identity switching issues [4], severely limiting the accuracy of sustained tracking and recognition for small targets. In practical applications like long-range vehicle tracking in traffic surveillance or ball trajectory analysis in sports events, small targets not only require high recognition accuracy [5] but also demand stable, long-term tracking in complex environments. Therefore, researching continuous tracking and recognition methods for small targets in video streams holds significant practical importance.

For instance, Rathore et al. utilized the Hough wavelet transform to extract small-target features, feeding them as input samples to a support vector machine to generate tracking and recognition results [6]. However, the Hough wavelet transform relies on traditional handcrafted features, which struggle to capture the complex semantic information of small targets and are susceptible to noise interference in complex backgrounds. Muzammul et al. employed a lightweight network to extract coarse-grained regions of interest (ROIs) within images. They enhanced the processing of ROI images through a dynamic adaptive guided object inference slicing mechanism to extract small-target features, ultimately outputting small-target tracking and recognition results [7]. Lightweight networks may miss extremely small targets, leading to biased segmentation regions that compromise subsequent feature extraction accuracy. Memon et al. optimized the dynamic weights of a comprehensive trajectory segmentation algorithm using target trajectory likelihood ratios and target recognition probabilities to generate candidate trajectories. An adaptive threshold mechanism then filtered these candidates to produce final tracking results [8]. Comprehensive trajectory segmentation algorithms typically process each frame’s trajectory fragments independently, failing to leverage temporal consistency in target motion. This approach risks losing tracking continuity when targets briefly disappear or become occluded. Das et al. employed a particle filter algorithm with imperialist competition to optimize parameters in a cooperative steering target tracking model for target tracking and recognition [9]. However, prolonged tracking may cause particle diversity to diminish, leading to target drift. Saini et al. employed a pixel attention detector to extract target features at different scales. Through a rotation detector, they processed these features to output target recognition results [10]. Pixel attention primarily focuses on salient regions—larger targets—and may overlook minute objects, reducing recognition accuracy for small targets.

To further enhance tracking and recognition of small objects in video streams, this study proposes a continuous tracking and recognition method for small video stream objects based on YOLO and a spatio-temporal context memory network. The YOLO series has garnered significant attention since 2016, with multiple iterations including YOLOv3, YOLOv5, YOLOv8, and YOLOv11. Among these, YOLOv8 achieves high detection speed while maintaining high accuracy, offering strong model scalability [11], ease of deployment and application, and demonstrating high robustness and stability across various complex scenarios. The Spatio-Temporal Context Memory Network (STCM-Net) preserves spatiotemporal contextual memory for objects. When objects are partially occluded, it restores tracking through memory matching, enhancing tracking recognition stability. To this end, this study proposes a continuous small object tracking and recognition method for video streams based on YOLO and the Spatio-Temporal Context Memory Network, improving recognition accuracy.

Based on the aforementioned analysis, the primary contribution of this paper is not the introduction of a brand-new fundamental network module. Instead, it focuses on addressing core challenges in small-target tracking and recognition within video streams, such as feature loss and interruption due to occlusion. This is achieved through systematic improvements and multi-component collaborative optimization of the YOLOv8 framework, resulting in a comprehensive and efficient solution for continuous tracking and recognition of small targets. Specifically, the unique contributions of this paper are reflected in the following three aspects: Firstly, a backbone feature extraction structure that integrates wavelet pooling and hybrid attention is designed. The WPConv module [12] decomposes the visual features of small targets into low-frequency global contours and high-frequency edge details through two-dimensional discrete wavelet transform, effectively suppressing aliasing effects in complex backgrounds. Meanwhile, the CBAM module [13] adaptively enhances the feature response in significant regions of small targets across both channel and spatial dimensions. The synergistic effect of these two components significantly improves the feature recognizability of small targets in low signal-to-noise ratio environments. Secondly, for the first time, the Spatio-Temporal Context Memory Network (STCM-Net) [14] and Bi-directional Feature Pyramid Network (BiFPN) [15] are jointly introduced in the neck network. The gated mechanism of ConvLSTM maintains dynamic spatiotemporal memory of targets across consecutive frames, utilizing historical information to compensate for missing features in the current frame when targets are partially occluded. The bidirectional weighted fusion strategy of BiFPN further aggregates multi-frame and multi-scale features, addressing the shortcoming of traditional feature pyramids that tend to attenuate small-target information. Thirdly, to address the instability of small-target bounding box regression, an Inner-PIoU loss function is designed. Through an adaptive penalty factor and an auxiliary bounding box scaling mechanism, it suppresses the expansion of predicted boxes while enhancing the localization accuracy of small targets at different scales. These improvements are not simply a stacking of existing technologies, but rather targeted coupling and adaptation around the main line of “feature enhancement—spatiotemporal memory—precise localization”, forming a unique technical path distinct from conventional YOLO improvement methods. This provides a new solution for continuous tracking and recognition of small targets in video streams.

## 2. Continuous Tracking and Recognition of Small Objects in Video Streams

### 2.1. Composition of the YOLO-Based Small Object Continuous Recognition Framework with Spatio-Temporal Context Memory Network

This paper proposes a small-object continuous recognition framework based on YOLO and a spatiotemporal context memory network to address the issues of low pixel proportion, easy feature loss, and complex background in video streams. This framework takes video streams as input and achieves continuous recognition through the collaborative linkage of the backbone network, neck network, and head network. In the backbone network, the wavelet pooling module (WPConv) is used to suppress aliasing effects and improve the ability to extract target features; Combining mixed attention (CBAM) to suppress environmental interference and improve tracking and recognition accuracy. The neck network introduces the Spatiotemporal Context Memory Network (STCM Net), which can still capture the semantic and dynamic spatiotemporal features of the target even when it is partially occluded, and adopts the Bidirectional Feature Pyramid (BiFPN) as the main feature fusion structure to compensate for the defect of feature loss. The design of the head network includes an Inner PIOU loss function to enhance the accuracy of bounding box localization and further improve the ability to track and recognize small targets. Through three-level structural collaboration, this framework effectively achieves continuous recognition of small targets in video streams.

**Figure 1:**
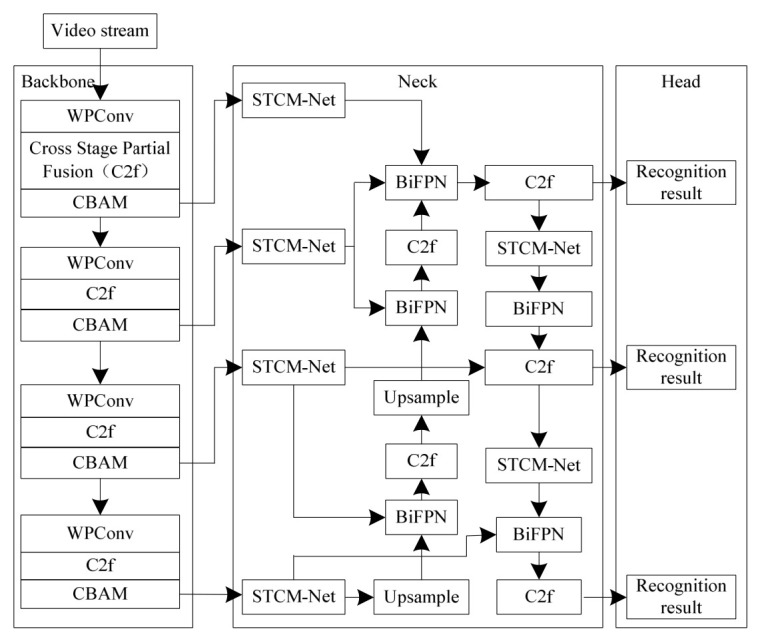
Recognition framework of YOLO and spatiotemporal context memory network model.

The small-target continuous recognition framework based on YOLO and spatiotemporal context memory network is shown in Figure 1.

### 2.2. Backbone Network Design

#### 2.2.1. Video Stream Small Object Feature Extraction via WPConv Module

To address the loss of critical small object features in video streams, the backbone network employs the WPConv module to hierarchically extract multi-scale visual features from small objects within each frame of the video stream while suppressing inter-frame noise interference. The WPConv module is a core component of the backbone network. Its design goal is to suppress the aliasing effect of small-target features during the convolution process and enhance the ability to extract multi-scale details. This module first employs a 3 × 3 convolution layer to extract local texture and edge information. Subsequently, it decomposes the features into a low-frequency approximation subband and a high-frequency detail subband through two-dimensional discrete wavelet transform, which correspond to the global contour of the target and the edge responses in the vertical, horizontal, and diagonal directions, respectively. Finally, the multi-scale fused features are reconstructed through wavelet inverse transform. Compared with traditional pooling operations, WPConv can retain high-frequency details and avoid the loss of small-target features during downsampling. The specific implementation process is as follows.

The convolutional layer within the WPConv module extracts initial visual features of small objects from a single frame image in the video stream. This process preserves the subtle textures of small objects in the video stream [16] while mitigating the initial interference from background noise. The formula for the basic visual features of small objects preliminarily output by the WPConv module is:

(1) $$x^{\prime }=B\left(C^{3\times 3}\left(x\right)\right)\cdot \varphi \left(B\left(C^{3\times 3}\left(x\right)\right)\right)$$

where $C^{3\times 3}$ is the convolution layer with a 3 × 3 kernel, used to extract local neighborhood features of small objects; B is the batch normalization operation, suppressing feature drift caused by inter-frame brightness and contrast variations in the video stream; φ is the Sigmoid activation function, enhancing the nonlinear discriminative power of small object features; $x^{\prime }$ is the preliminary output of the WPConv module for the basic visual features of small objects, preserving shallow-level details such as edges and textures.

Where $x^{\prime }$ is input into the WPConv module’s wavelet pooling layer. Through a two-dimensional discrete wavelet transform, features are decomposed into high-frequency detail subbands and low-frequency approximation subbands, achieving multi-scale decomposition of small object visual features. This simultaneously separates redundant information from complex background regions [17] and enhances high-frequency visual features of small object edge details. The calculation formula is:

(2) $$\left\{\begin{array}{c} X_{ll}=Lx^{\prime }L^{T}\\ X_{lh}=Hx^{\prime }L^{T}\\ X_{hl}=Lx^{\prime }H^{T}\\ X_{hh}=Hx^{\prime }H^{T} \end{array}\right.$$

where $X_{ll}$ represents the low-frequency approximation component (global outline of the small object); $X_{lh}$ denotes the vertical detail component (vertical edges of the small object); $X_{hl}$ indicates the horizontal detail component (horizontal edges of the small object); $X_{hh}$ signifies the diagonal/high-frequency noise component (corners of the small object); L and H are the low-pass and high-pass filters of the orthogonal wavelet, respectively; $L^{T}$ and $H^{T}$ are their transposes. The low-pass filter extracts the low-frequency global contours of small objects, while the high-pass filter preserves their high-frequency edge details. This decomposition effectively resolves the issue of small object details being obscured by complex video stream backgrounds.

The four subbands obtained by wavelet transform introduce key frequency-selective modulation links between the two. The low-frequency approximation component carries the main contour information of the target, and the module preserves and enhances it through learnable scaling parameters to ensure that the global structure is not distorted; And the three high-frequency detail components (vertical, horizontal, diagonal) correspond to different directions of edge and texture responses, respectively. For these high-frequency subbands, the module will use the gate weights generated by the previous convolutional layer, namely the output of the Sigmoid function in Formula (1), for dynamic amplitude modulation, to enhance the strong and stable high-frequency coefficients and highlight the subtle boundaries of small targets; Suppress false high-frequency activations caused by background textures, sudden changes in lighting, or sensor noise. Diagonal subbands often contain a large amount of noise, and the module introduces a soft threshold mechanism to selectively suppress the response in low signal-to-noise ratio areas without weakening the target corner information. After the above adaptive filtering, each subband carries more discriminative multi-scale information, and then performs inverse transformation to reconstruct the feature map, so that WPConv can not only preserve the spatial details of small targets, but also effectively suppress aliasing effects and environmental interference, significantly improving the robustness of subsequent feature extraction.

Perform a two-dimensional discrete wavelet inverse transform on $X_{ll}$, $X_{lh}$, $X_{lh}$, and $X_{hh}$ to reconstruct the visual feature Y, which encompasses multi-scale details of the small target. This completes the visual feature extraction in the WPConv module, calculated as follows:

(3) $$Y=L^{T}X_{ll}L+L^{T}X_{hl}H+H^{T}X_{lh}L+H^{T}X_{hh}H$$

The C2f module fuses multi-scale visual features from all levels Y to generate a fused multi-scale visual feature $Y^{\prime \prime }$ that incorporates rich details such as small object edges and textures while retaining high-level semantic information.

#### 2.2.2. Feature Response Enhancement in Important Pixel Regions Based on Hybrid Attention Module

The CBAM module is a lightweight serial attention structure composed of a Channel Attention Module (CAM) and a Spatial Attention Module (SAM). Its overall function is to adaptively recalibrate the channel and spatial weights of feature maps: CAM assigns importance coefficients to each channel by aggregating global context information, highlighting feature channels that are sensitive to small-target responses; SAM further learns the saliency of spatial locations on the feature maps enhanced by channels, strengthening the pixel regions where small targets are located and suppressing background noise. The two-stage cascade enables CBAM to significantly enhance the model’s ability to focus on key areas with minimal computational overhead. The specific calculation process is as follows: The fused multi-scale visual features $Y^{\prime \prime }$ extracted in Section 2.2.1 first pass through the CAM. This module aggregates channel-dimensional feature information via average pooling and max pooling operations, adaptively adjusts feature relationships between channels, and assigns higher weights to key feature channels. Element-wise multiplication then yields the channel-enhanced visual features. Subsequently, these features enter the SAM, which further strengthens feature responses in spatially important pixel regions. Through this two-stage collaborative enhancement, CBAM effectively boosts feature expressiveness [18], selectively focusing on task-relevant core features while reducing interference between targets and background. This enhances both the recognition capability and accuracy of YOLO and spatio-temporal context memory network models in identifying critical information [19].

For the input fused multi-scale visual feature $Y^{\prime \prime }$, after processing by the CAM, an intermediate multi-scale visual feature $Y_{1}^{\prime \prime }$ is first generated. Then, after processing by the SAM, a small-target weighted multi-scale visual feature $\tilde{Y}$ is output. The calculation formula is as follows:

(4) $$Y_{1}^{\prime \prime }=M_{c}\left(\begin{array}{c} Y^{\prime \prime } \end{array}\right)⊗Y^{\prime \prime }$$

(5) $$\tilde{Y}=Sigmoid\left(f\left[{AvgPool}_{channel}\left(Y_{1}^{\prime \prime }\right),\;{MaxPool}_{channel}\left(Y_{1}^{\prime \prime }\right)\right]\right)⊗Y_{1}^{\prime \prime }$$

where ⊗ denotes element-wise multiplication; $M_{c}\left(\begin{array}{c} Y^{\prime \prime } \end{array}\right)$ and $M_{s}\left(\begin{array}{c} Y_{1}^{\prime \prime } \end{array}\right)$ represent the attention maps in the channel and spatial directions, respectively. The calculation formula for $M_{c}\left(\begin{array}{c} Y^{\prime \prime } \end{array}\right)$ is as follows:

(6) $$\begin{array}{cc} M_{c}\left(Y^{\prime \prime }\right) & =Sigmoid\left(MLP\left(A\nu gPool\left(Y^{\prime \prime }\right)\right)+MLP\left(MaxPool\left(Y^{\prime \prime }\right)\right)\right)\\ & =Sigmoid\left(W_{1}\left(W_{0}\left({Y^{\prime \prime }}_{avg}^{c}\right)\right)+W_{1}\left(W_{0}\left({Y^{\prime \prime }}_{max}^{c}\right)\right)\right) \end{array}$$

where MLP is a multilayer perceptron and the MLP is a two-layer fully connected network. The number of neurons in the first layer is 1/16 of the number of input channels, and it employs a ReLU activation function. The number of neurons in the second layer is restored to the original number of channels.

$\;W_{0}$ and $W_{1}$ are parameter matrices for feature map weighting; ${Y^{\prime \prime }}_{avg}^{c}$ is the average pooling feature; ${Y^{\prime \prime }}_{max}^{c}$ is the max pooling feature.

The formula for calculating $M_{s}\left(\begin{array}{c} Y_{1}^{\prime \prime } \end{array}\right)$ is as follows:

(7) $$\begin{array}{c} M_{s}\left(Y_{1}^{\prime \prime }\right)=\varphi \left(F^{7\times 7}\left(\left[A\nu gPool\left(Y_{1}^{\prime \prime }\right);MaxPool\left(Y_{1}^{\prime \prime }\right)\right]\right)\right)\\ =\varphi \left(F^{7\times 7}\left(\left[{Y^{\prime \prime }}_{a\nu g}^{s};{Y^{\prime \prime }}_{max}^{s}\right]\right)\right) \end{array}$$

where φ denotes the Sigmoid activation function; $F^{7\times 7}$ represents the 7 × 7 convolution operation; ${Y^{\prime \prime }}_{a\nu g}^{s}$ denotes the average pooling feature; ${Y^{\prime \prime }}_{max}^{s}$ represents the max pooling feature.

### 2.3. Output of Small Object Continuous Recognition Results Based on Neck Network Video Streams

#### 2.3.1. Generating Multi-Scale Spatio-Temporal Features for Small Targets

The STCM-Net module is a spatiotemporal context memory module based on a convolutional long short-term memory network. Its design goal is to perform temporal modeling on the small-target features of consecutive frames in a video stream, capturing the motion dynamics and appearance evolution of the target. This enables the use of historical memory to compensate for the missing spatiotemporal information in the current frame when the target is partially occluded or temporarily disappears. The module takes ConvLSTM as its core unit, with the weighted multi-scale visual features of the current frame as its input, while also receiving the hidden state and memory unit passed from the previous frame. ConvLSTM controls the writing of new features, the retention of historical memory, and the generation of current temporal output through three gating mechanisms: the input gate, forget gate, and output gate. All operations are performed using convolutional operations rather than fully connected operations, thus preserving the spatial structure of the features. In each frame, ConvLSTM first calculates the input gate, forget gate, output gate, and candidate memory unit based on the current input and previous hidden state. Then, it updates the memory unit and outputs the hidden state of the current frame. This hidden state integrates historical spatiotemporal information with the visual features of the current frame and is sent to subsequent convolutional layers to generate multi-scale spatiotemporal features of small targets.

Therefore, to address the challenges of complex video backgrounds, low pixel coverage of small targets, and susceptibility to feature loss, the small-object-weighted multiscale visual features $\tilde{Y}$ are input into the STCM-Net module. This module learns the semantic and dynamic spatio-temporal representations of small objects across consecutive video frames, extracting multiscale spatio-temporal features [20]. Specifically, the STCM-Net module is designed using a convolutional long short-term memory (ConvLSTM) network to extract these multi-scale spatio-temporal features.

The fused multi-scale visual features ${\tilde{Y}}_{t}$ from the tth frame are input into the ConvLSTM unit of the STCM-Net module. Through a gating mechanism, it learns the spatio-temporal associations between small targets in the frame and historical frame features within the video stream. It sequentially computes the input gate, forget gate, output gate, and candidate memory units, as described by the following formula:

(8) $$r_{t}=\varphi \left(W_{r}\times \left({\tilde{Y}}_{t},Z_{t-1}\right)+b_{r}\right);$$

(9) $$q_{t}=\varphi \left(W_{q}\times \left({\tilde{Y}}_{t},Z_{t-1}\right)+b_{q}\right);$$

(10) $$o_{t}=\varphi \left(W_{o}\times \left({\tilde{Y}}_{t},Z_{t-1}\right)+b_{o}\right);$$

(11) $$C_{t}=\tanh\left(W_{c}\times \left({\tilde{Y}}_{t},Z_{t-1}\right)+b_{c}\right),$$

where $r_{t}$ represents the small object features in the video stream output by the input gate, focusing on valid small object features while suppressing background noise; $q_{t}$ represents the small object features in the video stream output by the forget gate, discarding irrelevant historical background information while preserving the temporal characteristics of small objects; $o_{t}$ represents the small object features in the video stream output by the output gate, filtering information relevant to the spatio-temporal representation of small objects; $C_{t}$ represents candidate memory unit features, storing spatio-temporal information of multi-scale features fused for small objects in the current frame; $Z_{t-1}$ denotes the hidden state of frame t−1; $W_{r}$, $W_{q}$, $W_{o}$, and $W_{c}$ represent the weights for the input gate, forget gate, output gate, and candidate memory unit, respectively; $b_{r}$, $b_{q}$, $b_{o}$, and $b_{c}$ denote the corresponding biases; ∗ denotes the convolution operator.

Update the memory units of ConvLSTM based on $r_{t}$, $C_{t}$, and $q_{t}$ to store and transmit short-term spatio-temporal context information, as expressed by the following formula:

(12) $${\hat{Q}}_{t}=q_{t}⊙{\hat{Q}}_{t-1}+r_{t}⊙C_{t}$$

Among these, ${\hat{Q}}_{t}$ serves as the memory unit for frame t, storing the core spatio-temporal context information of small targets in frame t of the video stream. It integrates historical memory with current frame features to resolve feature discontinuities caused by small-target occlusions. ${\hat{Q}}_{t-1}$ functions as the memory unit for frame t−1 of the video stream, preserving the historical spatio-temporal memory of small targets to provide temporal support for updating current frame features.

Based on $o_{t}$ and ${\hat{C}}_{t}$, the hidden state for frame t is computed as follows:

(13) $$Z_{t}=o_{t}⊙\tanh({\hat{C}}_{t})$$

where $Z_{t}$ represents the hidden state of frame t, containing the spatio-temporal context representation after small object updates.

First, ${\tilde{Y}}_{t}$ undergoes convolution processing to generate the initial spatio-temporal prior $Z_{0}^{m}$. Then, ${\tilde{Y}}_{t}$ is concatenated with $Z_{t}$ and fed into the STCM-Net module. By integrating current frame features with historical spatio-temporal information, the model ultimately generates multi-scale spatio-temporal features for small objects, as shown in the following formula:

(14) $$Z_{0}^{m}=F\left({\tilde{Y}}_{t}\right)$$

(15) $${\hat{Y}}_{t}=Z_{0}^{m}⊗\theta \left(p({\tilde{Y}}_{t},Z_{t})\right)$$

where θ denotes STCM-Net; F represents the convolution operation; p signifies the concatenation operation; ${\hat{Y}}_{t}$ denotes the small-object multi-scale spatio-temporal features.

#### 2.3.2. Feature Generation at Different Scales in Bidirectional Feature Pyramid Network

The BiFPN module is a bidirectional feature pyramid structure designed to fuse spatiotemporal features of different scales from the backbone network and STCM-Net. The main design ideas of this module include three points: First, nodes with only one input edge are removed to simplify the computational graph and reduce redundancy; second, two bidirectional information flow paths, top-down and bottom-up, are added to achieve efficient interaction between high-level semantic features and low-level detail features; third, learnable dynamic weights are introduced to fuse different input features based on their importance [21,22]. Compared with traditional PANet, BiFPN can more effectively preserve the shallow details of small targets while avoiding the dilution of deep features on small-target responses. Through multiple iterative fusions, the multi-scale features output by BiFPN exhibit stronger context awareness. The specific implementation is as follows.

The BiFPN module employs a bidirectional information transfer mechanism (coordinating top-down and bottom-up processes) to prune marginal nodes with weak feature fusion capabilities. This enhances the network’s expressive power and information transfer efficiency while reducing redundancy [23]. Simultaneously, it introduces a learnable dynamic weighting mechanism that adaptively adjusts the importance weights of different input features. This fully leverages the contextual information and complementarity across feature scales, achieving lightweight implementation of both YOLO and spatio-temporal context memory network models while improving the quality of multi-scale spatial feature fusion.

The dynamic weighted fusion formula for BiFPN is as follows:

(16) $$V=\sum_{i=1}^{N}\frac{\omega_{i}}{\varepsilon +\sum_{j=1}^{N}\omega_{j}}\cdot {\hat{Z}}_{i}$$

where V represents the spatiotemporal features of small objects after weighted fusion; ${\hat{Z}}_{i}$ denotes the i th input multi-scale spatial feature; N represents the maximum value of the number of input multi-scale spatiotemporal features; $\omega_{i}$ and $\omega_{j}$ denote distinct learnable weight parameters; ε is a minimal constant preventing division by zero to ensure computational stability. The C2f module performs cross-stage refinement fusion on V, filtering complex background interference in video streams to output multi-scale features $\hat{V}$ containing both shallow details and deep semantic information of small objects.

### 2.4. Head Network Loss Function Design

After feeding the $\hat{V}$ obtained in Section 2.3.2 into the head network, the detection branch first generates small object prediction bounding boxes [24,25]. The Inner-PIoU loss function is then introduced to optimize bounding box regression accuracy, addressing issues like bounding box drift and slow convergence in video streams. This ultimately outputs high-precision continuous recognition results for small objects in video streams.

Based on the $\hat{V}$, the head network generates the predicted bounding box ${\hat{\beta }}_{t}$ and the ground truth bounding box $\beta_{t}^{\prime }$ for small objects in the video stream’s frame t. It then extracts core geometric parameters to serve as the foundation for loss calculation. Specifically, the detected bounding box has width ${\hat{w}}_{t}$ and height ${\hat{h}}_{t}$, while the ground truth bounding box has width $w_{t}^{\prime }$ and height $h_{t}^{\prime }$. The absolute distances between corresponding edges of the detected and ground truth boxes are $dw_{1}$, $dw_{2}$, $dh_{1}$, and $dh_{2}$ respectively. These parameters are decoded from the $\hat{V}$ via the head regression branch, directly relating to the spatial position and scale information of the small object.

To suppress overfitting of small object bounding boxes during regression, an adaptive penalty factor λ is calculated based on the intrinsic dimensions of the ground truth bounding boxes [26]. This prevents convergence slowdown caused by penalties unrelated to the minimum bounding box size, as expressed by the following formula:

(17) $$\lambda =\left(\frac{dw_{1}}{w_{t}^{\prime }}+\frac{dw_{2}}{w_{t}^{\prime }}+\frac{dh_{1}}{h_{t}^{\prime }}+\frac{dh_{2}}{h_{t}^{\prime }}\right)/4$$

Based on λ, the quality penalty function $\xi \left(\lambda \right)$ for recognition bounding boxes of small objects in video streams is constructed as follows:

(18) $$\xi \left(\lambda \right)=1-e^{-\lambda^{2}}$$

Combining the CIoU (Complete Intersection over Union) [27] regression metric $R_{\mathrm{CIoU}}$ with $\xi \left(\lambda \right)$, we define the PIoU (Probability Intersection over Union) regression metric $R_{\mathrm{PIoU}}$ for small targets in video streams, as follows:

(19) $$R_{\mathrm{PIoU}}=R_{\mathrm{CIoU}}-\xi \left(\lambda \right)$$

Based on $R_{\mathrm{PIoU}}$, define the PIoU loss function $L_{\mathrm{PIoU}}$ as the base loss for small object detection bounding box regression, formulated as follows:

(20) $$L_{\mathrm{PIoU}}=1-R_{\mathrm{PIoU}}$$

To address the limited adaptability of PIoU for small objects at different scales, a scaling factor α is introduced to generate auxiliary bounding boxes and compute the Inner-IoU metric, defined as follows:

(21) $$\chi_{t,r}^{\prime }=x_{t,r}+\frac{w_{t}^{\prime }\cdot \alpha }{2};$$

(22) $$\chi_{t,l}^{\prime }=x_{t,l}-\frac{w_{t}^{\prime }\cdot \alpha }{2};$$

(23) $$\chi_{t,b}^{\prime }=y_{t,b}+\frac{h_{t}^{\prime }\cdot \alpha }{2};$$

(24) $$\chi_{t,t}^{\prime }=y_{t,t}-\frac{h_{t}^{\prime }\cdot \alpha }{2};$$

(25) $${\hat{\chi }}_{r}=x_{p,c}+\frac{{\hat{w}}_{t}\cdot \alpha }{2};$$

(26) $${\hat{\chi }}_{l}=y_{p,c}-\frac{{\hat{w}}_{t}\cdot \alpha }{2};$$

(27) $$\gamma^{\prime }=\left[\min(\chi_{t,r}^{\prime },{\hat{\chi }}_{r})-\max(\chi_{t,l}^{\prime },{\hat{\chi }}_{l})\right]\cdot \left[\min(\chi_{t,b}^{\prime },{\hat{\chi }}_{b})-\max(\chi_{t,t}^{\prime },{\hat{\chi }}_{t})\right];$$

(28) $$\gamma^{\prime \prime }=(w^{\prime }\cdot h^{\prime })\cdot \alpha^{2}+(\hat{w}\cdot \hat{h})\cdot \alpha^{2}-\gamma^{\prime };$$

(29) $$R_{\mathrm{Inner}-\mathrm{IoU}}=\frac{\gamma^{\prime }}{\gamma^{\prime \prime }},$$

where $\chi_{t,r}^{\prime }$ and $\chi_{t,l}^{\prime }$ denote the right and left boundary coordinates of the auxiliary bounding box for the small object’s ground truth bounding box in frame t of the video stream; $\chi_{t,b}^{\prime }$ and $\chi_{t,t}^{\prime }$ denote the bottom and top boundary coordinates of the auxiliary bounding box for the small object’s ground truth bounding box in frame t of the video stream; ${\hat{\chi }}_{r}$ and ${\hat{\chi }}_{l}$ denote the right and left boundary coordinates of the auxiliary bounding box for the small object’s recognition bounding box in frame t of the video stream; $x_{t,r}$ and $x_{t,l}$ is the center abscissa of the right and left boundaries of the real target box of the small target in frame t of the video stream; $y_{t,b}$ and $y_{t,t}$ is the center vertical coordinate of the lower and upper boundaries of the real target box of the small target in frame t of the video stream. $x_{p,c}$, $y_{p,c}$ represent the center coordinates of the small object detection bounding box in frame t of the video stream;$\;\;\gamma^{\prime }$ denotes the intersection area of the small object auxiliary bounding box in frame t of the video stream; $\gamma^{\prime \prime }$ indicates the union area of the small object auxiliary bounding box in frame t of the video stream; $R_{\mathrm{Inner}-\mathrm{IoU}}$ signifies the Inner-IoU metric for small objects in the video stream; ${\hat{\chi }}_{b}$ and ${\hat{\chi }}_{t}$ denote the lower and upper coordinates of the detection bounding box auxiliary boundary.

(30) $${\hat{\chi }}_{b}=y_{t,b}-\frac{h}{2}$$

(31) $${\hat{\chi }}_{t}=y_{t,t}-\frac{h}{2}$$

where h is the height of the bounding box.

By integrating PIoU loss and Inner-IoU metrics, the final Inner-PIoU loss function is defined as follows:

(32) $$L_{\mathrm{Inner}-\mathrm{PIoU}}=L_{\mathrm{Base}-\mathrm{PIoU}}+{\lambda \cdot R}_{\mathrm{CIoU}}+R_{\mathrm{Inner}-\mathrm{IoU}}$$

where $L_{\mathrm{Base}-\mathrm{PIoU}}$ represents the original PIoU loss, retaining only the basic regression constraints on the position and size of the bounding box, and does not contain any offset logic related to RCIoU internally; λ represents an adaptive weight, dynamically adjusting the penalty intensity based on the edge deviation of the bounding box.

Compared with other IoU losses, the core difference in this loss lies in two points: firstly, it introduces an adaptive penalty factor λ based on the margin deviation between the predicted box and the real box, which enables the loss to perceive the uncertainty of bounding box regression and impose additional constraints when the prediction deviation is large; The second is to dynamically adjust the gradient scale through the auxiliary box mechanism of Inner IoU, while the existing IoU loss is calculated using a fixed scale, which makes it difficult to simultaneously consider the regression accuracy of targets of different sizes. These two points together constitute the differentiated contribution of Inner PIOU compared with existing loss functions. The head network continuously optimizes the decoding weights of the YOLO and spatio-temporal context memory network models through backpropagation based on the $L_{\mathrm{Inner}-\mathrm{PIoU}}$ [28]. It ultimately outputs the category probabilities for small objects in frames t of the video stream and the optimized bounding box coordinates, yielding the continuous recognition results ${\tilde{Z}}_{t}$ for small objects in the video stream.

### 2.5. Implementation of Continuous Small Object Tracking and Recognition in Video Streams

Based on the continuous recognition results of small objects output in Section 2.4, combined with Uniformly Accelerated Kalman Filtering (UAKF) [29] and the Deep Simple Online Tracking (DeepSORT) algorithm [30], continuous tracking and recognition of small objects in the video stream are achieved.

Using the uniform acceleration Kalman filter algorithm, process ${\tilde{Z}}_{t}$ to predict small object tracking recognition boxes:

(33) $$D_{t}=\tau D_{t-1}+K_{t}({\tilde{Z}}_{t}-\rho \tau D_{t-1})$$

where $D_{t}$ is the small object tracking box at frame t; $K_{t}$ is the Kalman gain; $D_{t-1}$ is the predicted state vector of the small object at frame t−1; τ is the state transition matrix; and ρ is the observation matrix.

The DeepSORT algorithm calculates the Mahalanobis distance to measure the similarity of motion information between the predicted small-target tracking recognition box and the recognized target box, as follows:

(34) $$l_{1}={\left({\tilde{Z}}_{i^{\prime }}-D_{i^{\prime }}\right)}^{T}\Sigma_{i^{\prime }}^{-1}\left({\tilde{Z}}_{i^{\prime }}-D_{i^{\prime }}\right)$$

where $D_{i^{\prime }}$ and ${\tilde{Z}}_{i^{\prime }}$ represent the $i^{\prime }$th predicted small-target tracking bounding box and the recognized target bounding box, respectively; $\Sigma_{i^{\prime }}$ denotes the standard deviation matrix between small-target trajectories.

The minimum cosine distance is used to measure the similarity of appearance information between the predicted tracking recognition box and the recognition target box, as expressed by the following formula:

(35) $$l_{2}=\min\left\{1-\delta_{{\tilde{Z}}_{i^{\prime }}}\delta_{D_{i^{\prime }}}\right\}$$

where $l_{2}$ represents the cosine distance between the prediction recognition box and the target box, $\delta_{D_{i^{\prime }}}$ and $\delta_{{\tilde{Z}}_{i^{\prime }}}$ correspond to the appearance feature vectors of the small-target tracking recognition box and the recognition target box for the K-th prediction, respectively.

The matching cost for continuous small object tracking recognition in video streams is:

(36) $$\sigma =\varpi l_{1}+\left(1-\varpi \right)l_{2}$$

where ϖ is the adjustment factor.

The Hungarian algorithm is applied frame-by-frame to select matching pairs (predicted tracking recognition boxes and recognition target boxes) where σ falls below the matching threshold. Successfully matched predicted tracking recognition boxes update their state while maintaining their original trajectory, enabling continuous tracking recognition of small objects in video streams.

## 3. Experimental Analysis

The UAVDT (UAV Detection and Tracking) dataset used in this article is one of the widely used public benchmark datasets in the field of object detection and tracking from the perspective of unmanned aerial vehicles. It was jointly released by the University of Bristol and other institutions in the UK, and is mainly used to evaluate the detection and continuous tracking capabilities of low-altitude unmanned aerial vehicle platforms for ground small targets in complex urban scenes. Since its publication, this dataset has been adopted by multiple studies in the fields of computer vision and intelligent perception at home and abroad as a standardized testing platform for algorithm validation, with high authority and comparability. The distribution of target size relative to image size in this dataset is concentrated below 0.1, with small targets being the main focus, which is consistent with the research problem set in this paper. In all experiments in this article, the input images were uniformly scaled to 640 × 640 pixels. At this scale, targets with relative sizes less than 0.1 correspond to absolute pixel sizes mainly distributed between 32 × 32 and 64 × 64, which conforms to the general definition of small targets in the international academic community. Therefore, this dataset is highly compatible with the small-target continuous tracking and recognition problem studied in this paper, and can effectively test the performance of the algorithm under challenges such as sparse features, cluttered background, and target occlusion. To ensure the reproducibility and fairness of experimental results, this study strictly followed the official partitioning protocol of the dataset during model training and testing, without any additional screening or modification of the original data. All experiments were conducted on a unified hardware platform and with the same hyperparameter settings. Its basic information is shown in Table 1.

**Figure 2:**
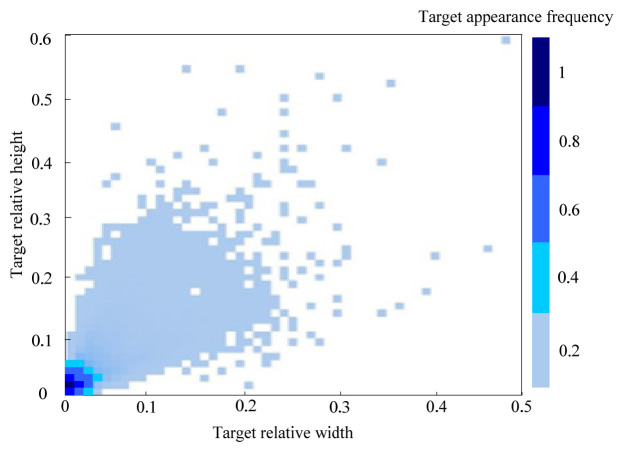
Distribution of target relative sizes.

The normalized distribution of target relative sizes (target size relative to image size) in the dataset is illustrated in Figure 2. The relative dimensions in Figure 2 are macro distribution indicators. The commonly used definition of small targets in the field of object detection is mainly based on absolute pixel size. For example, the COCO standard considers targets smaller than 32 × 32 pixels as small targets. In this study, all video frames were uniformly scaled to 640 × 640 pixels before being sent to the network. Under this uniform input scale, the absolute pixel size corresponding to targets with relative sizes less than 0.1 in Figure 2 mainly falls within the range of 32 × 32 to 64 × 64, with most falling below 64 × 64, which meets the commonly used criteria for defining small targets.

As shown in Figure 2, high-frequency target regions are concentrated in the lower-left corner, i.e., within the range of relative sizes less than 0.1, indicating that small targets dominate the dataset.

During the model training process, all experiments utilize the same hyperparameter settings to ensure reproducibility. The input video frames are uniformly scaled to 640 × 640 pixels, and the batch size is set to 16. The optimizer employs stochastic gradient descent, with an initial learning rate set at 0.01. A cosine annealing strategy is adopted for learning rate decay, with a weight decay coefficient of 0.0005 and a momentum factor of 0.937. The total number of training epochs is 300, with the first three epochs utilizing a warm-up strategy where the warm-up learning rate linearly increases from 0.001 to 0.01. In terms of data augmentation, random horizontal flipping, random scaling, mosaic enhancement, and mixed enhancement techniques are employed to enhance the model’s generalization ability for small targets. In the loss function, the bounding box regression part utilizes the Inner-PIoU loss proposed in this paper, the classification loss employs binary cross-entropy loss, and the confidence loss utilizes focal loss. The model saves weights every 10 epochs during training, and the model with the highest MOTA on the validation set is selected as the final test model. Under the aforementioned hyperparameter settings, the loss function tends to converge after approximately 150 epochs, without experiencing overfitting or oscillation, indicating a stable and effective training process.

**Figure 3:**
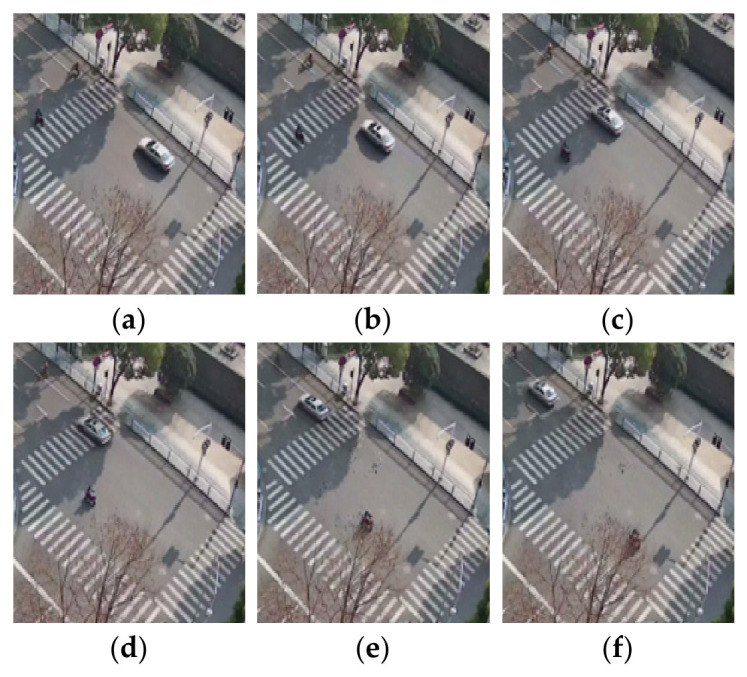
Sample frames from the original video stream. (**a**) 1st frame; (**b**) 3rd frame; (**c**) 5th frame; (**d**) 7th frame; (**e**) 10th frame; (**f**) 12th frame.

**Figure 4:**
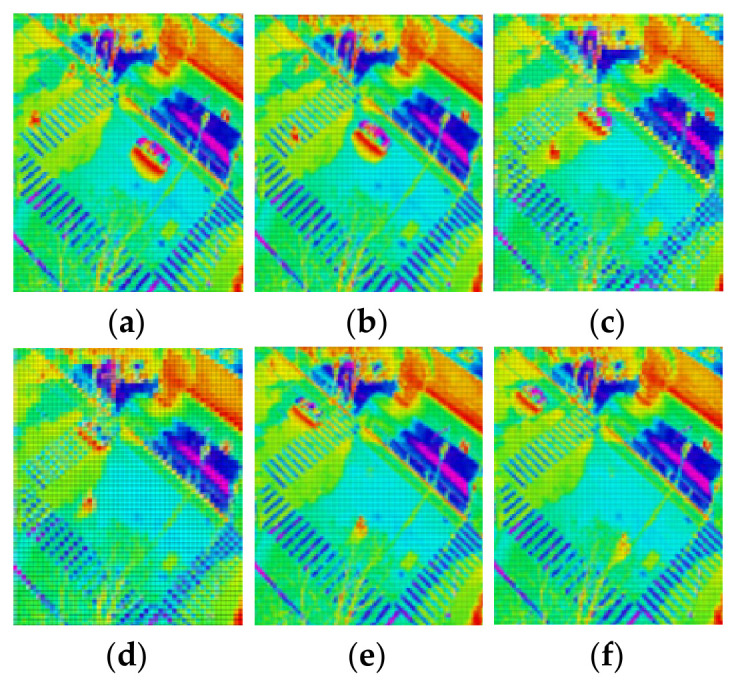
Feature extraction results of small targets in video stream. (**a**) 1st frame; (**b**) 3rd frame; (**c**) 5th frame; (**d**) 7th frame; (**e**) 10th frame; (**f**) 12th frame.

**Figure 5:**
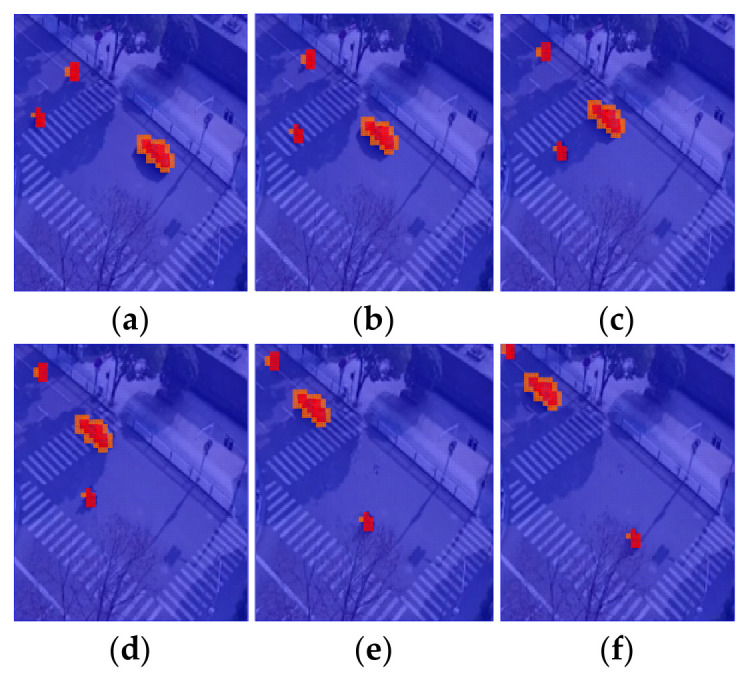
Spatiotemporal feature extraction results of small targets in video stream. (**a**) 1st frame; (**b**) 3rd frame; (**c**) 5th frame; (**d**) 7th frame; (**e**) 10th frame; (**f**) 12th frame.

In this dataset, a video stream is randomly selected, and the original video stream is shown in Figure 3. The spatiotemporal features of small targets are extracted using the method proposed in this paper, and the results of spatial feature extraction are shown in Figure 4 and Figure 5.

Figure 3a–f display the image content of six consecutive frames (frames 1, 3, 5, 7, 10, 12) from the original video stream, serving as the visual representation of the input data for our method. The background contains complex elements such as buildings, roads, and vegetation. Each frame within the original video stream contains three small moving objects. These objects are distributed relatively widely across the images, not concentrated in a single area, and all occupy a small proportion of pixels within the frames. Consequently, tracking and identifying these small objects presents a high level of complexity. Additionally, varying degrees of occlusion of the small objects are present in frames 10 and 12. This video stream encompasses typical challenging scenarios—multiple targets, small dimensions, dynamic motion, and partial occlusion—making it suitable for validating the robustness and effectiveness of the proposed method in complex video streams.

As shown in Figure 4a–f, the regions corresponding to the three small targets exhibit significant feature activation responses in each frame, indicating that the WPConv module effectively extracts visual features of small targets. Weaker feature responses in non-target areas such as buildings and vegetation demonstrate that the CBAM module successfully suppresses irrelevant information, enhancing the distinction between targets and background. Despite the small target size, feature responses remain stable across different frames without loss or blurring due to scale changes, demonstrating WPConv’s robust multiscale feature extraction capability. High consistency is observed in feature maps across frames, with no noticeable inter-frame jitter or noise artifacts, indicating that the batch normalization and wavelet reconstruction mechanisms in WPConv effectively suppress illumination variations and random noise in the video stream.

Figure 5a–f demonstrate that our method effectively utilizes the spatiotemporal context memory network to analyze small-target visual features, learning their semantic and dynamic spatiotemporal representations within the video stream to extract spatiotemporal characteristics of small targets. Background noise is further reduced, indicating that the spatio-temporal context memory network effectively integrates temporal information and enhances spatio-temporal consistency in the target region. From Frame 1 to Frame 12, the feature responses of each small target exhibit smooth, continuous spatial variations, demonstrating that the ConvLSTM unit successfully models the motion dynamics and temporal dependencies of small targets. The spatio-temporal features of small objects exhibit no noticeable jitter or drift during motion, demonstrating the superiority of the spatio-temporal context memory network in motion smoothness and spatio-temporal alignment. In frames 10 and 12, despite partial occlusion of the electric vehicle target, its spatio-temporal feature responses remain clear and accurately positioned, with no significant feature loss or misactivation. This demonstrates that the spatio-temporal context memory network, through the coordinated action of the forget gate and input gate, suppresses irrelevant background information during occlusion while utilizing historical memory to complete missing features in the current frame, highlighting its effectiveness in recovering from short-term occlusions.

**Figure 6:**
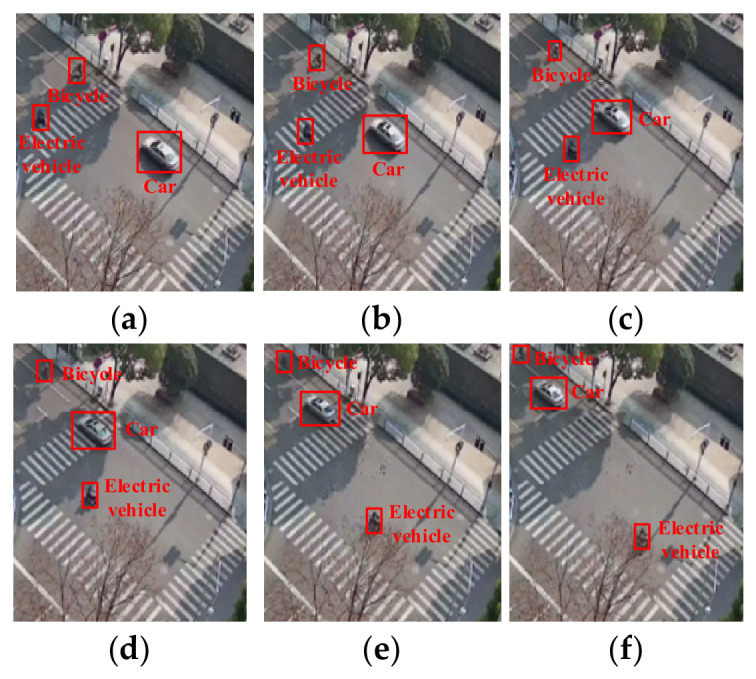
Continuous tracking and recognition results of small targets in the video stream. (**a**) 1st frame; (**b**) 3rd frame; (**c**) 5th frame; (**d**) 7th frame; (**e**) 10th frame; (**f**) 12th frame.

The small-target continuous tracking and recognition results obtained using the proposed method on this video stream are shown in Figure 6.

From Figure 6a–f, it can be observed that the proposed method effectively achieves continuous tracking and recognition of small targets. Each frame contains three categories of small targets: automobiles, electric vehicles, and bicycles. This demonstrates that the head network possesses robust classification capabilities based on feature extraction and spatio-temporal modeling. No significant offset or excessive/insufficient bounding box size was observed, indicating that the Inner-PIoU loss function effectively optimizes bounding box regression accuracy, particularly enhancing small-target localization. From Frame 1 to Frame 12, the tracking boxes for all three targets remained continuous without any small-target loss, demonstrating the stable performance of DeepSORT combined with UAVKF in data association and state prediction. In frames 10 and 12, the small electric vehicle target was obscured by trees, yet its tracking box remained stable with reasonable position and size. No drift toward the occluding object or background was observed, demonstrating that the spatio-temporal features and memory mechanism provided by STCM-Net enabled our method to maintain target state during occlusion and achieve precise tracking and recognition.

The continuous tracking accuracy of small targets using our method is measured by MOTA (Mean Object Tracking Accuracy). This indicator comprehensively takes into account three error sources: missed detection, false detection, and identity tag switching. It is used to evaluate the tracking system’s ability to maintain the continuity and accuracy of the target trajectory throughout the entire video sequence. Where MOTA closer to 1 indicates higher tracking accuracy. MOTP (Mean Object Tracking Precision) represents the average metric between all successfully matched predicted small-target tracking boxes and their corresponding boxes. This indicator measures the average alignment degree between all successfully matched predicted bounding boxes and the corresponding real target boxes, using the overlap degree of bounding boxes or position errors as the measurement basis. MOTP values closer to 1 indicate higher tracking accuracy.

The calculation formula for MOTA is as follows:

(37) $$MOTA=1-\frac{FN+FP+S_{1}}{G}$$

In the formula, FN represents the total number of times small targets that actually exist have not been tracked and recognized. FP represents the total number of false small targets that were incorrectly identified as non-existent. $S_{1}$ represents the total number of times the identity tag of the same real target undergoes incorrect switching during continuous tracking. G represents the total number of occurrences of all real small targets in a video sequence.

The calculation formula for MOTP is as follows:

(38) $$MOTP=\frac{\sum_{t,i}d_{t,i}}{\sum_{t}c_{t}}$$

In the formula, $d_{t,i}$ represents the overlap metric between the i-th successfully matched predicted small-target tracking recognition box and the corresponding real annotated recognition box in the t-th frame. $\sum_{t,i}d_{t,i}$ represents the cumulative sum of the overlap metrics between all successfully matched predicted boxes and the true boxes. $c_{t}$ represents the number of successfully matched small-target pairs in the t-th frame. $\sum_{t}c_{t}$ represents the total number of successful matching pairs in the entire video sequence.

To validate the effectiveness of our improvements to the YOLOv8n model, ablation experiments were conducted using YOLOv8n as the baseline, evaluating the introduction of WPConv, CBAM, STCM-Net, BiFPN, and the Inner-PIoU loss function. The results are shown in Table 2.

Analysis of Table 2 reveals that for Method 1, YOLOv8n exhibits low MOTA and MOTP values for small-object continuous tracking and recognition, indicating poor tracking accuracy. For Method 2, introducing WPConv into YOLOv8n significantly improves MOTA by 0.1 and MOTP by 0.05, demonstrating that the WPConv module substantially enhances feature representation capabilities for small objects. For Method 3, incorporating CBAM further improved both MOTA and MOTP values, enhancing small-target continuous tracking recognition accuracy. This demonstrates that the CBAM module enables the YOLOv8n model to focus on key regions, suppress background noise, and improve feature signal-to-noise ratio and discriminative power. For Method 4, incorporating STCM-Net elevated MOTA and MOTP to 0.94 and 0.93, respectively, approaching ideal values. This indicates high small-object tracking accuracy, demonstrating STCM-Net’s ability to enhance YOLOv8n’s modeling of occlusion and motion continuity, significantly improving tracking persistence. For Method 5, incorporating BiFPN resulted in a modest increase of 0.03 for both MOTA and MOTP. This demonstrates BiFPN’s ability to strengthen feature interaction and improve feature integrity for small targets in complex scenes. For Method 6, after introducing the Inner-PIoU loss function, MOTA and MOTP reached 0.99 and 0.98, respectively, both very close to the ideal value of 1. This indicates extremely high recognition accuracy for persistent tracking of small targets.

**Figure 7:**
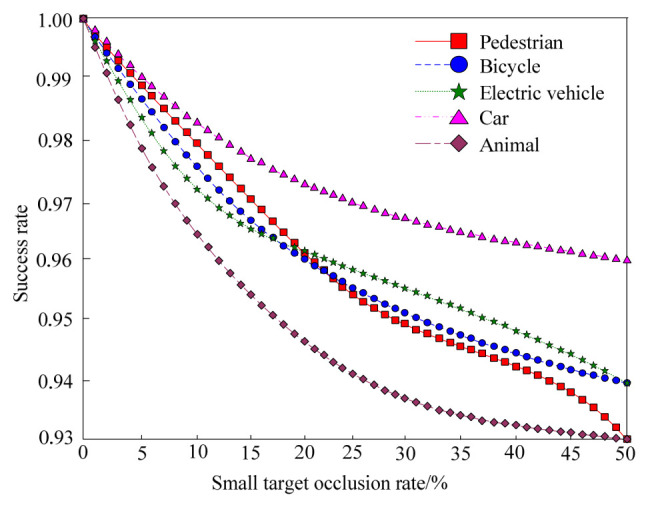
Analysis results of success rate of continuous tracking and recognition of small targets.

The success rate is used to analyze the accuracy of small-target continuous tracking and recognition in the method proposed in this paper. The success rate refers to the proportion of frames where small targets are correctly and continuously tracked and recognized, out of the total number of frames where the target appears in the video stream. Its value ranges from 0 to 1, with a higher value indicating higher accuracy of small-target continuous tracking and recognition. The success rate of small-target continuous tracking and recognition using the method proposed in this paper is analyzed under different small-target occlusion probabilities. Figure 7 shows the success rate of continuous tracking and recognition for small targets under different occlusion probabilities.

As shown in Figure 7, the success rate of continuous tracking and recognition for all small-target types decreases as the occlusion rate increases. The varying degrees of decline indicate that the inherent characteristics of small targets significantly impact their occlusion robustness. Vehicles exhibit the smallest decrease in success rate, primarily due to their relatively large size, regular structure, smooth motion, and prominent features, which minimize the impact of occlusion. Animals show the largest decrease in success rate, attributed to their small size, irregular motion, and unstable features, making them most sensitive to occlusion. The minimum success rates for pedestrians, bicycles, electric vehicles, and animals are approximately 0.93, 0.94, 0.94, 0.96, and 0.93, respectively. Pedestrians and animals occupy a smaller proportion of pixels in video stream frames, resulting in lower continuous tracking recognition success rates. However, their minimum success rate of 0.93 is very close to the ideal value of 1, indicating that the proposed method achieves high accuracy in continuous tracking recognition of small targets under varying occlusion rates.

To verify the generalization ability of the proposed method on different video data, in addition to the aforementioned self-built dataset, two publicly available small-target video datasets were additionally selected for testing: VisDrone2019 and OTA100. VisDrone2019 is a database of drone aerial perspectives, containing dense small targets such as pedestrians and vehicles. OTA100 includes various scenes with occlusion and illumination variations. The Multiple Object Tracking Accuracy (MOTA) and Multiple Object Tracking Precision (MOTP) of each dataset for the traditional YOLOv8n and the improved YOLOv8n model in this paper are shown in Table 3.

As can be seen from Table 3, the method proposed in this paper achieves MOTA and MOTP values above 0.94 on three video datasets from different sources and scenarios, showing significant improvement compared with the baseline model. Especially in VisDrone2019, which features a drone perspective with high density of small targets and complex movements, the proposed method maintains high tracking and recognition accuracy, demonstrating the good cross-scenario generalization ability of the combination of WPConv and STCM-Net. The OTA100 dataset contains many occlusions, and the MOTA value of the proposed method reaches 0.94, further verifying the robustness of the spatiotemporal memory mechanism to occlusions.

**Figure 8:**
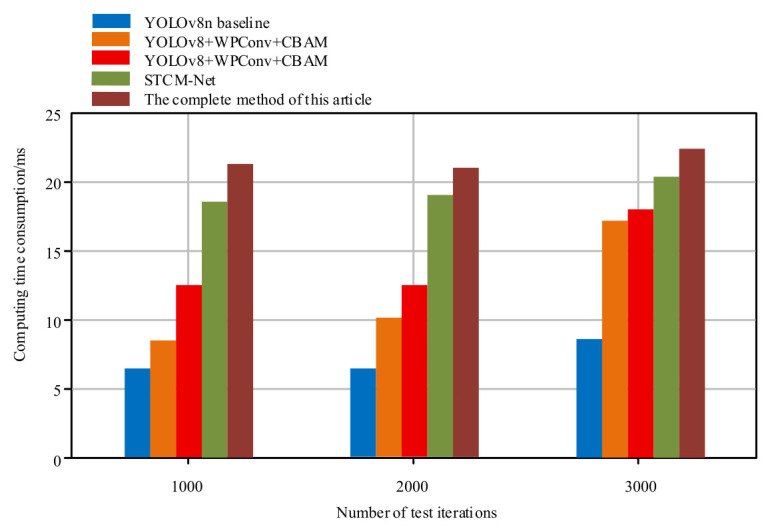
Comparison of computational efficiency among different methods.

In addition to accuracy, computational efficiency is a key metric for real-time tracking and recognition of video streams. To evaluate the computational efficiency of the proposed method, tests were conducted on a unified hardware platform with video streams of input resolution 1920 × 1080 and batch size 1. The average computation time for different methods was recorded. The comparative methods included the YOLOv8n baseline, YOLOv8 + WPConv + CBAM, YOLOv8 + WPConv + CBAM + STCM-Net, and the complete method proposed in this paper. The results are shown in Figure 8.

According to Figure 8, as the number of test iterations increases, the computation time for each method shows an upward trend, which is in line with expectations. Among all the compared methods, the YOLOv8n baseline consistently maintains the lowest computation time, approximately 6–9 ms, demonstrating the fastest inference speed. YOLOv8 + WPConv + CBAM increases the time by approximately 2–3 ms compared with the baseline, indicating that the introduction of attention mechanisms and lightweight convolutions brings about a certain computational overhead. The time consumption of YOLOv8 + WPConv + CBAM + STCM-Net further increases to around 18–20 ms. This suggests that although the STCM-Net module may enhance feature extraction capabilities, it also significantly increases the computational burden on the model. The complete method proposed in this paper, as the final solution, has the highest time consumption. This indicates that in pursuit of higher accuracy or more complex feature fusion, researchers have made certain sacrifices in terms of computational efficiency. Nevertheless, the complete method in this paper can still maintain a time consumption of around 23 ms after 3000 iterations, which remains highly practical for processing video streams with a resolution of 1920 × 1080.

To objectively evaluate the progressiveness of the method proposed in this paper, three representative existing methods were selected for comparative experiments. The visual tracking system based on extreme learning machine proposed in the literature [6] extracts target features using Haar wavelet transform and achieves small-target tracking and recognition through extreme learning machine. The small-target detection method based on guided object inference slicing proposed in the literature [7] utilizes a lightweight network to extract coarse-grained regions of interest and enhances small-target features through a dynamic adaptive slicing mechanism. The multi-target tracking data association method based on integrated trajectory splitting filtering proposed in the literature [8] employs a comprehensive trajectory segmentation algorithm to generate candidate trajectories and utilizes a gated threshold adaptive mechanism to filter target trajectories. These three methods represent different technical routes such as traditional machine learning, lightweight deep learning detection, and trajectory association optimization, respectively, and are closely related to the task of continuous small-target tracking and recognition. They are suitable as benchmarks for comparing the method proposed in this paper.

The comparative experiments were conducted on the UAVDT dataset, selecting 30 video sequences with the highest proportion of small targets as the test set. The total number of video frames is approximately 12,000, with a resolution of 1920 × 1080. All comparative methods were run on a unified hardware platform, employing the same input preprocessing and post-processing strategies. Evaluation metrics included Multi-Object Tracking Accuracy (MOTA), Multi-Object Tracking Precision (MOTP), Sustained Small-Target Tracking and Recognition Success Rate, and Average Inference Speed. Among them, the definitions of MOTA and MOTP are consistent with those in the ablation experiments. The success rate is defined as the ratio of the number of frames where targets are correctly tracked and recognized to the total number of frames where targets appear. The average inference speed is measured in frames per second. The replication of each comparative method follows the public description in the original paper, and parameter adaptation is performed for the same small-target tracking task. The experimental results are shown in Table 4.

As can be seen from Table 4, the method proposed in this paper significantly outperforms all comparative methods in terms of three accuracy indicators: MOTA, MOTP, and success rate. Although the method in reference [6] exhibits robustness in small-target tracking, the Haave wavelet transform, as a handcrafted feature, struggles to capture the complex semantic information of small targets. This limits its performance in the cluttered background of the UAVDT dataset, achieving an MOTA of only 0.68 and a success rate of 0.71. The method in reference [7] enhances the detectability of small targets through a dynamic slicing mechanism, achieving an inference speed of 45 FPS, indicating good real-time performance. However, its lightweight network tends to miss small targets, resulting in an MOTA of 0.71, which is still lower than the 0.99 achieved by the method proposed in this paper. The method in reference [8] focuses on multi-target data association and has certain advantages in occlusion scenarios. However, its comprehensive trajectory segmentation algorithm processes each frame of trajectory segments independently, without fully utilizing temporal motion consistency, leading to poor tracking continuity and an MOTA of 0.65. Although the YOLOv8n baseline has a significant speed advantage, reaching 86 FPS, its accuracy indicators are far lower than those of the method proposed in this paper. The method proposed in this paper leads in accuracy while maintaining an inference speed of 52 FPS, which is higher than the 25 to 30 FPS required for real-time processing of video streams, achieving a good balance between accuracy and efficiency.

The method proposed in this paper integrates modules such as WPConv, CBAM, STCM-Net, BiFPN, and Inner-PIoU on the YOLOv8n baseline. WPConv increases the computational overhead of feature decomposition and reconstruction through wavelet pooling operations. CBAM’s channel and spatial attention mechanism introduces additional global pooling and convolution operations. The convolutional long short-term memory network in STCM-Net significantly increases computational complexity due to the concatenation of multiple convolutional layers in the gating mechanism. BiFPN further increases the computational load of feature interaction through cross-scale weighted fusion. The superposition of these modules extends the overall forward propagation time of the model compared with the baseline, thereby reducing the real-time frame-processing rate of the video stream. However, thanks to the efficient backbone design of YOLOv8n itself and the lightweight implementation of each module during the inference stage, the frame-processing rate of the complete model can still be stably maintained at over 50 frames per second, which is higher than the threshold of 25 to 30 frames per second required for real-time processing of video streams, and meets the continuous tracking and recognition requirements in practical applications.

## 4. Conclusions and Discussion on Future Work

To enhance the accuracy of small-target continuous tracking and recognition in complex backgrounds, this study proposes a video stream-based small-target continuous tracking and recognition method integrating YOLO and spatio-temporal context memory networks. By introducing WPConv into the YOLOv8n network, the method suppresses aliasing effects in small targets, improving feature extraction capabilities. CBAM is employed to suppress irrelevant information in video streams, reducing mutual interference between small-target recognition and the surrounding environment. STCM-Net maintains spatio-temporal context memory for small targets, enabling the capture of semantic and dynamic spatio-temporal features even when partially occluded. BiFPN suppresses the weakening or loss of small object feature information. The Inner-PIoU loss function enhances feature extraction and precise bounding box localization, thereby improving small object continuous tracking and recognition accuracy. Experimental results demonstrate that, compared with the YOLOv8n baseline model, the improved YOLOv8n model achieves a MOTA of 0.99 (up from 0.72) and a MOTP of 0.98 (up from 0.81), significantly enhancing continuous tracking and recognition capabilities for small objects in video streams.

Although the method presented in this paper has achieved excellent performance in tracking and recognizing small targets in various scenarios and has a computational efficiency that meets real-time requirements, there are still areas for improvement: (1) Currently, when there is extreme occlusion (occlusion rate > 80%) or when the target disappears and reappears, the update of temporal-spatial memory may result in cumulative errors. Subsequently, a memory enhancement mechanism or graph neural network can be introduced to model the temporal-spatial relationships between targets. (2) The current experiments focus on visible light video streams. In the future, this method can be extended to multimodal data such as infrared and millimeter-wave radars, and the use of multi-sensor fusion can be utilized to enhance the ability of small-target tracking and recognition in night or adverse weather conditions. (3) Edge deployment and online learning are also important directions. By introducing incremental learning strategies, the model can continuously adapt to new scenarios in practical applications without the need for complete re-training.

## Figures and Tables

**Table 1:** Basic information of video streaming dataset.

Project	Value/Description
Total number of video sequences	79 of them
Total frame rate	Approximately 33,488 frames
Average frame rate	25FPS
Resolution range	960 × 540 to 3840 × 2160
Number of annotated target categories	5 categories (pedestrians, bicycles, electric vehicles, cars, animals)

**Table 2:** Results of ablation experiment analysis.

Method Number	YOLOv8n	WPConv	CBAM	STCM-Net	BiFPN	Inner-PIoU	MOTA	MOTP
1	√	×	×	×	×	×	0.72	0.81
2	√	√	×	×	×	×	0.82	0.86
3	√	√	√	×	×	×	0.89	0.90
4	√	√	√	√	×	×	0.94	0.93
5	√	√	√	√	√	×	0.97	0.96
6	√	√	√	√	√	√	0.99	0.98

**Table 3:** MOTA and MOTP on different datasets.

Methods	Self-Built Dataset	VisDrone2019	OTA100
YOLOv8n (MOTA/MOTP)	0.72/0.81	0.68/0.78	0.65/0.75
The method presented in this article (MOTA/MOTP)	0.99/0.98	0.96/0.95	0.94/0.93

**Table 4:** Performance comparison between the proposed method and existing benchmark methods.

Methodology	MOTA	MOTP	Success Rate	Inference Speed/FPS
Method described in the literature [6]	0.68	0.75	0.71	18
Method described in the literature [7]	0.71	0.79	0.74	45
Method described in the literature [8]	0.65	0.73	0.69	22
YOLOv8n baseline	0.72	0.81	0.73	86
Proposed Method	0.99	0.98	0.96	52

## Data Availability

All data are included in the article.
